# Metastatic progression of breast cancer along with decreased mitochondrial cell death priming of breast cancer cells: a case report

**DOI:** 10.1093/omcr/omae014

**Published:** 2024-03-25

**Authors:** Yeliz Aka, Hulya Ozdemir, Nese Torun, Filiz Aka Bolat, Ozgur Kutuk

**Affiliations:** Department of Immunology, Baskent University School of Medicine, Adana Dr. Turgut Noyan Medical and Research Center, Adana, Turkey; Department of Medical Biology, Baskent University School of Medicine, Ankara, Turkey; Department of Radiology, Baskent University School of Medicine, Adana Dr. Turgut Noyan Medical and Research Center, Adana, Turkey; Department of Nuclear Medicine, Baskent University School of Medicine, Adana Dr. Turgut Noyan Medical and Research Center, Adana, Turkey; Department of Pathology, Baskent University School of Medicine, Adana Dr. Turgut Noyan Medical and Research Center, Adana, Turkey; Department of Immunology, Baskent University School of Medicine, Adana Dr. Turgut Noyan Medical and Research Center, Adana, Turkey

## Abstract

Metastatic breast cancer remains to be a major cause of cancer-related deaths in women. Exploring the molecular mechanisms to identify targetable alterations in progressing breast cancer and developing functional tools to predict therapy response in these patients are needed. In this report, we present a case of breast cancer patient who progressed following surgery and adjuvant endocrine therapy. Radiological and pathological analyses revealed metastasis to liver and brain. Paired liquid biopsies demonstrated acquired ERBB2 mutations in addition to TP53 and PIK3CA mutations, which were also present before progression. BH3 profiling test demonstrated decreased mitochondrial cell death priming in CTCs of the patient after progression. In conclusion, novel personalized treatment strategies are needed to monitor metastatic breast cancer patients for better clinical benefit.

## INTRODUCTION

Breast cancer is the most frequently diagnosed malignancy and second common reason in terms of cancer-related death in women [1]. In fact, evaluation of circulating tumor DNA (ctDNA) and circulating tumor cells (CTCs) in metastatic breast cancer patients provided important insight into how tumors evolve in the patient [2]. BH3 profiling is a novel technology that has been shown to determine mitochondrial cell death priming and predict response to chemotherapy in clinical oncology settings [3]. Herein we report a case of progressed metastatic breast cancer with acquired ERBB2 mutations and decreased mitochondrial cell death priming.

## CASE REPORT

**Figure 1:**
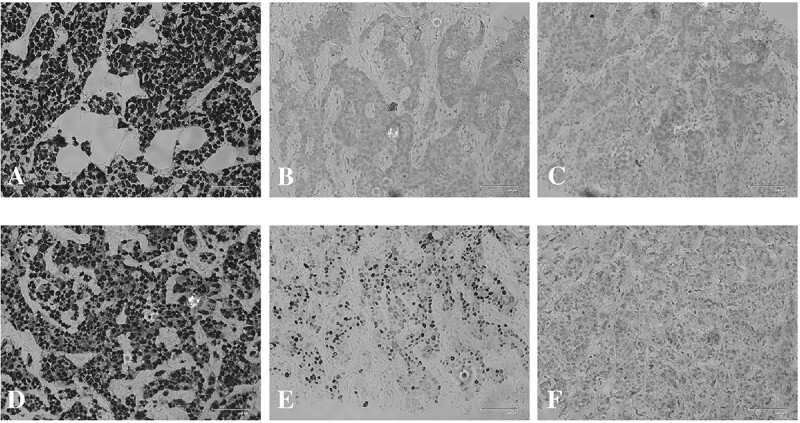
Immunohistochemistry of the primary tumor for (**A**) ER, (**B**) PR and (**C**) HER2 and of the liver metastasis for (**C**) ER, (**D**) PR and (**E**) HER2.

The patient with breast cancer presented with unilateral mammary carcinoma and lymph node metastases at age 65. Patient received neo-adjuvant chemotherapy (cyclophosphamide plus epirubicin, docetaxel) and underwent modified-radical mastectomy. First blood collection for CTC isolation and ctDNA analysis was performed before surgery. Histopathological analysis of the primary tumor revealed grade 2 invasive ductal carcinoma with lymphovascular and perineural invasion. Tumor tissue was evaluated as estrogen receptor (ER) positive, progesterone receptor (PR) and HER2 negative (Fig. 1). HER2 was also found negative by means of FISH assay in the primary tumor tissue.

**Figure 2:**
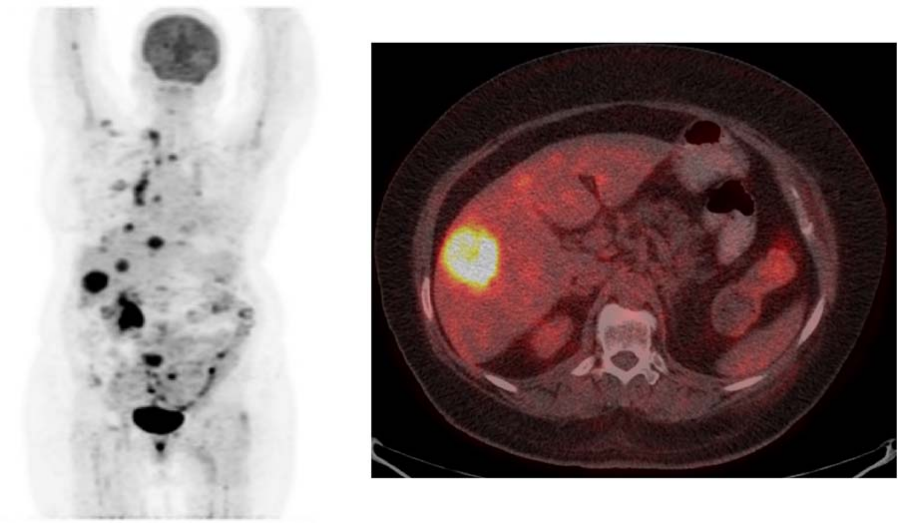
Whole-body maximum-intensity-projection (MIP) PET showed abnormal FDG uptake in the right infraclavicular area, mediastinal lymph nodes, liver and skeletal system suggesting widespread metastasis in the patient. Axial section of the liver on fused PET-CT showed abnormal intense FDG uptake (SUVmax: 15.8).

No distant metastases were detected by PET-CT and USG screens. Circulating tumor cells (CTCs) were isolated by using EasySep Direct Human CTC Enrichment Kit (Vancouver, BC, Canada). Cells propagated in DMEM/F12 (ThermoFisher Scientific, Carlsbad, CA, USA) supplemented with 10% heat-inactivated FBS (Sigma, St Louis, MO, USA), 100 IU/ml penicillin, and 100 μg/ml streptomycin (ThermoFisher Scientific, Carlsbad, CA, USA) in a humidified incubator at 37°C and 5% CO_2_ using AlgiMatrix 24-well plates as oncospheroids. Microplate-based BH3 profiling was done as described before [3]. ctDNA (circulating tumor DNA) mutation analysis by FoundationOne Liquid CDX detected PIK3CA H1047R (MAF:1.3) and TP53 R248Q (MAF:8.6) mutations. No primary tumor tissue sample was available for targeted sequencing. The patient received aromatase inhibitor letrozole and fosamax for 17 months until metastasis was detected in the liver by PET-CT (Fig. 2).

**Figure 3:**
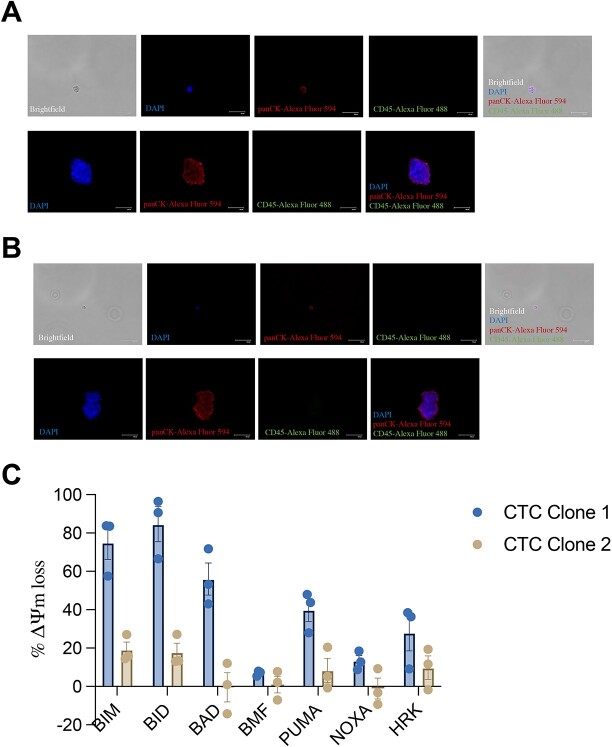
CTCs were isolated by using EasySep Direct Human CTC Enrichment Kit. Isolated single CTCs and 3D oncospheroids were stained with DAPI, panCK-Alexa Fluor 594 and CD45-Alexa Fluor 488 and cells were imaged with EVOS FLoid digital microscopy system. Scale bars, 100 μm. Representative images of cells (A) before progression (CTC Clone 1) and (**B**) after progression (CTC Clone 2) are shown. (**C**) BH3 profiling was performed on CTC oncospheroids before and after progression. Graphs represent the means of three independent experiments ± SEM.

Following USG-guided FNAC, histopathological evaluation of liver metastasis revealed ER positive, PR positive and receptor and HER2 negative atypical epithelial breast cancer cells with hyperchromatic nucleus, enlarged cytoplasm and prominent nucleolus (Fig. 1). ERBB2 FISH was also negative for the metastatic tissue in the liver. We collected the second set of blood samples for CTC and ctDNA analysis before liver biopsy. ctDNA mutation analysis by FoundationOne Liquid CDX reported PIK3CA H1047R (MAF:4.1), TP53 R248Q (MAF:22.5), ERBB2 V777L (MAF:20.2) and ERBB2 R713Q (MAF:0.17) mutations. Furthermore, targeted next-generation sequencing of tissue samples from locally relapsed tumor and liver metastases lesions indicated concordant mutation profiles in PIK3CA, TP53 and ERBB2. Of note, the patient was reported BRCA1/2-negative in germline and tumor hotspot sequencing data. BH3 profiling of CTC oncospheroids before and after progression demonstrated significantly decreased mitochondrial cell death priming in circulating breast cancer cells after progression (Fig. 3).

**Figure 4:**
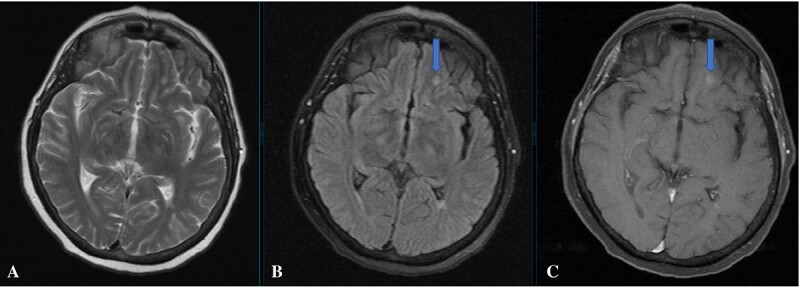
A metastatic nodular lesion was detected in the left frontobasal subcortical white matter which was hyperintense on axial T2-weighted MRI (**A**) and FLAIR image (**B**). The lesion also shows contrast enhancement (**C**). Blue arrows indicate lesions.

Accordingly, the endocrine therapy was switched to cisplatin+ gemcitabine+zoledronic acid. The patient progressed and the therapy was further switched to paclitaxel+zoledronic acid. Palliative cranial RT was administered after brain metastases were detected in the patient by MRI (Fig. 4).

The patient died shortly afterwards due to complications related to brain metastases and metabolically progressed disease.

## DISCUSSION

Tumor evolution during progression of the metastatic breast cancer is usually accompanied by emerging clones of cancer cells with additional mutations in various oncogenes including ERBB2 and resistance to chemotherapy [4]. In fact, liquid biopsy has been shown to be an effective tool to monitor disease progression and tumor response to treatment in breast cancer patients [5, 6]. The patient we described herein presented with liver and brain metastasis after modified-radical mastectomy and adjuvant chemotherapy. By using paired liquid biopsies before and after progression, we demonstrated that the patient acquired additional ERBB2 V777L and R713Q mutations. ERBB2 V777L mutation has been reported before and it was shown to confer resistance to targeted therapies including trastuzumab and neratinib [7, 8]. However, the functional consequence of ERBB2 R713Q mutation remains to be identified. Immunohistochemical analysis liver metastasis biopsy sample revealed that the tumor becomes PR-positive, but still no amplification of ERBB2 was detected. Comprehensive cancer cell profiling to personalize therapy for each patient or to identify emerging clones of therapy-resistant cells and cancer progression is inevitable. BH3 profiling has been shown to predict sensitivity or resistance to various conventional chemotherapeutics and targeted therapies in clinical setting [3, 9, 10]. We determined the mitochondrial priming of paired CTC oncospheroids before and after patient progression and we clearly demonstrated that mitochondria of CTCs after progression of the patient were less primed with a higher apoptotic threshold, indicating emerging chemoresistance. Further studies are essential to advance our understanding how metastatic progression in breast cancer by means of genetic and functional precision medicine technologies.

## Data Availability

The data generated to support the findings are available within the article.
